# Cloning and characterization of low-temperature adapted GH5-CBM3 endo-cellulase from *Bacillus subtilis* 1AJ3 and their application in the saccharification of switchgrass and coffee grounds

**DOI:** 10.1186/s13568-020-00975-y

**Published:** 2020-03-05

**Authors:** Lingling Ma, Rakhmanova Aizhan, Xin Wang, Yanglei Yi, Yuanyuan Shan, Bianfang Liu, Yuan Zhou, Xin Lü

**Affiliations:** Lab of Bioresources, College of Food Science and Engineering, Northwest A&F University, Yangling, 712100 Shaanxi China

**Keywords:** *B. subtilis*, Endoglucanase, Clone and expression, GH5-CBM3, Lignocellulose

## Abstract

Endocellulase is a key cellulase for cellulosic material pretreatment in the industry by hydrolyzing long cellulose chains into short chains. To investigate the endocellulase characteristics from *Bacillus subtilis* 1AJ3, and increase its production yield, this paper cloned an endocellulase gene denoted *CEL*-5A from strain 1AJ3 and expressed in *E. coli* BL21 (DE3). The *CEL*-5A gene was sequenced with a full-length of 1500 bp, encoding a totally of 500 amino acids, and containing two domains: the GH5 family catalytic domain (CD) and the CBM3 family cellulose-binding domain (CBD). Recombinant endocellulase Cel-5A with a His-tag was purified of the Ni-NTA column, and SDS-PAGE results demonstrated that Cel-5A exhibited a molecular weight of 56.4 kDa. The maximum enzyme activity of Cel-5A was observed at pH 4.5 and 50 °C. Moreover, it was active over the broad temperature region of 30–60 °C, and stable within the pH range of 4.5–10.0. In addition, Co^2+^ was able to increase enzyme activity, while the majority of metal ions demonstrated stable enzyme activity under low- concentration. The substrate specificity of Cel-5A exhibited a high specific activity on the β-1,3-1,4 glucan linkage from barley. The Michaelis–Menten constant and the maximum velocity of the recombinant Cel-5A for CMC-Na were determined as 14.87 mg/mL and 19.19 μmol/min/mg, respectively. When Cel-5A was applied to the switchgrass and coffee grounds, its color became lighter and the biomass was observed to loosen following hydrolyzation. The saccharification rate reached 12% of the total weight of switchgrass in 20 h. These properties highlight the potential application of Cel-5A as an endocellulase in the pretreatment of biomass, for example, in the coffee grounds/waste, and related industries.

## Introduction

Cellulose, the major component of biomass, could be hydrolyzed into reducing sugars by cellulases, and can subsequently converted into ethanol and other chemicals (Hasunuma et al. 2013). Over the past few decades, a great deal of attention has been focused on the cellulose hydrolysis bio-technique due to the urgent need of green energy. Microbial hydrolyzation is one of the principle techniques used to convert cellulose into reducing sugars. Much research has been performed on cellulases originating from fungi, bacteria, and yeasts. Cellulases are divided into three types based on differences in structure, functions and processes: endo-glucanases (EC 3.2.1.4), exo-glucanases (EC 3.2.1.91), and β-glucosidases (EC 3.2.1.21). These three cellulases work synergistically to convert cellulose into fermentable sugars. Endo-cellulase makes an important contribution to cellulose hydrolyzation, as it focuses on the hydrolyzation of β-1,4-glucosidic glucan linkages, resulting in the conversion of long cellulose chains into short chains. This allows a greater amount of terminality exposed in the cellulose chains, providing a larger action site for the process of exo-cellulase hydrolyzation. Lastly, cellobiose and oligocellulose are hydrolyzed into glucose by β-glucosidases for further fermentation by yeasts or for the transfer into other chemicals. The glycoside hydrolase family is the classification of glycoside hydrolases based on the glycosidic bond molecular mechanism and the Carbohydrate Active Enzymes (CAZy) database (Lombard et al. 2014). Endo-cellulase are generally distributed in GH5-10, 12, 26, 44, 45, 48, 51, 74, and 124. In particular, GH5 is the largest family containing endo-β-1,4-glucanase, endo-β-1,4-xylanase, β-glucosidase, β-mannosidase, lichenase etc., and has been detected in numerous species (e.g. plants, animals, bacteria, archaea, eukaryotes and viruses) (Nguyen et al. 2018).

Temperature and pH are the main driving factors of enzyme activity, and thus also decide their application fields. A wide pH range is required for the application of enzymes in numerous conditions. Moreover, thermal- (over 60 °C) and low- (30–50 °C) temperatures are obstacles for enzyme applications in the industry, with a need for enzymes that have a wide temperature adaption ability, particularly for high enzyme activity at low temperature, which can simultaneously save energy. Enzymes adapted to low-temperatures have a wide temperature adaptability, ranging from low to thermal temperatures. They are also advantageous in terms of saving energy and interacting with other enzymes, such as lignin-degrading enzymes (Lin et al. 2018) at 20–30 °C and xylanases (Chang et al. 2017; Liu et al. 2019) at 50–60 °C. Moreover, it has been reported that cellulase exhibit minimal adsorption on lignin under 30 °C, therefore cellulase could free from cellulose substrate and reduced enzyme activity loss (Zanchetta et al. 2018). Thus, enzymes adapted to low-temperatures are able to combine and cooperate with other enzymes in more applications compared to other enzyme types. In addition, the cloning and characterization of low-temperature enzymes from microbes have also been reported (Dhar et al. 2015; Garsoux et al. 2004; Yang and Dang 2011), while it has also been observed that decreasing the cellulase hydrolyzation temperature can promote the fusion of the saccharification and fermentation processes in the bioethanol production industry (Crespim et al. 2016).

High yielding industrial productions requires high density initial material. However, the mixing or stirring of such material is generally difficult, thus enabling the material to “flow” is an urgent problem that needs to be solved. For example, endo-cellulase has already been used in the pulp industry (Li et al. 2018; Verma et al. 2016), where long cellulose chains are converted into shorter chains by randomly breaking glucan linkages. In particular, biomass lignocellulose is generally cut into pieces, which is equivalent to pretreatment following enzyme hydrolysis.

In the current paper, the cellulolytic bacterium *B. subtilis* 1AJ3 was isolated from rotten wood obtained from Qinling Mountains (China) under 37 °C. It was found that strain 1AJ3 can grow in an acid environment. We identified a GH5 family CD and a CBM3 family CBD in the whole ORF endo-cellulase (*CEL*-5A) sequence. Homology modeling was applied to reveal its structure, concluding a GH5 family classical TIM-like structure, with CBM3 forming a beta sandwich with a jelly roll topology. Moreover, the purified recombinant enzyme Cel-5A was obtained using a His-tag, and related recombinant enzyme properties were measured. The application of Cel-5A to switchgrass and coffee grounds raw materials demonstrated its potential application in hydrolyze unpretreated biomass.

## Materials and methods

### Materials

*Bacillus subtilis* 1AJ3 (GenBank No. MG062801), a cellulolytic bacterium isolated from rotten wood obtained from Qinling Mountains (China) (Ma et al. 2020), which was used as the clone template in this study. An analysis of the 16S rRNA gene sequence of strain 1AJ3 shared 100% sequence identity with the 16S rRNA gene of *B. subtilis* strain JCM 1465, *B. subtilis* strain NBRC 13719, and *B. subtilis* strain DSM 10 in the NCBI (National Center for Biotechnology Information) database. Strain 1AJ3 was cultured and stored in LB culture medium (NaCl 10 g/L, Tryptone 10 g/L, Yeast extract powder 5 g/L). It was maintained in a 25% glycerin tube with a bacterial suspension in LB medium at − 20 °C. The LB medium added to the final concentration of 100 μg/mL kanamycin was the culture medium of *Escherichia coli* BL21 containing the recombinant vector. All media were used after sterilization at 121 °C for 20 min.

The chemicals used for this study were obtained from Sigma-Aldrich (St. Louis, MO, USA) and were of analytical grads.

### Influence of culture medium pH on accumulating reducing sugar content

The single carbon source medium with different initial pH values (2–10) was adjust using HCl and NaOH. After culturing at 37 °C using a rotary shaker (180 rpm) for 48 h, the supernatant of the culture was collected by centrifugation. The reducing sugar content was measured via the DNS method (Miller 1959).

### Recombinant plasmid construction of endocellulase gene

A cellulase gene, *CEL*-5A, from *B. subtilis* 1AJ3 was amplified from the genomic DNA via a polymerase chain reaction (PCR). The genomic DNA of *B. subtilis* 1AJ3 was extracted using a bacterial DNA Kit (Omega Bio-tek, Inc.), and subsequently used as a template of cellulase cloning. The gene encoding of the cellulase was amplified using primers designed based on the cellulase gene sequence of the strain *B. velezensis* JTYP2 (GenBank: CP020375.1), whereby “endoglucanase” was identified from NCBI. The entire *CEL*-5A open reading frame (ORF) was amplified from the template of the strain genomic DNA via PCR using the forward primer, 5′-CATG*CCATGG*GCATGAAACGGTCAATTTCTATT-3′ (*Nco*I site italic) and the reverse primer, 5′-CCG*CTCGAG*ATTGGGTTCTGTTCCCCAAA-3′ (*Xho*I site italic). The PCR products and the pET-28a (+) vector (Novagen, Germany) were subject to double endonuclease digestion and linked through the *Nco*I and *Xho*I sites using the restriction enzymes (Takara Bio, Shiga, Japan). The recombined plasmid was detected correctly by 1% agarose gel electrophoresis and transferred into *E. coli* BL21 (DE3) for cloning and expression. The amplification of the recombinant plasmids was performed using the universal primer T7/T7er (5′-TAATACGACTCACTATAGGG-3′ and 5′-GCTAGTTATTGCTCA GCGG-3′), and the PCR products were verified via sequencing in order to obtain the whole ORF sequence of *CEL*-5A. Finally, the purified recombinant enzyme Cel-5A had a 6× his tag at the C-terminus.

### Expression and purification of the recombinant enzyme Cel-5A in *E. coli* BL21

The bacterium containing recombinant Cel-5A was cultured in LB liquid medium supplemented with 100 μg/mL Kan on a rotary shaker (180 rpm) at 37 °C. The production of the recombinant Cel-5A was induced with 0.2 mM of isopropyl-β-thiogalactopyranoside (IPTG) at an OD_600nm_ of 0.6 at 37 °C for 5 h.

Bacterium culture with recombinant Cel-5A was collected by centrifugation at 10,000×*g* for 10 min at 4 °C and washed with 1 × PBS buffer solution (pH 7.2) twice. Cells were re-suspended in 1 × PBS buffer solution, followed by ultrasonication for 20 min using a SCIENTZ-IID ultrasonic homogenizer (Ningbo Scientz Biotechnology Polytron Technologies Inc., Zhejiang, China). The cell debris was removed by centrifugation at 15,000×*g* for 20 min at 4 °C. The supernatant was collected and applied to a Ni-NTA affinity chromatography column (GE Healthcare Bio-Sciences AB, Uppsala, Sweden). Hybrid protein was then washed away with 40 mM imidazole and the recombinant eluted of Cel-5A protein with his-tag with 80 mM imidazole made up in 1 × PBS buffer solution (pH 7.2). The eluted protein was detected with 12% SDS-PAGE.

### Cellulase activity assay

Enzyme activity toward CMC was measured according to the method of Ghose (Ghose 1987), whereby the reducing sugars concentrations were determined via a DNS method (Miller 1959). The reaction mixture was composed of 100 μL of 1.0% CMC in 50 mM citrate buffer (pH 4.5), 50 μL of enzyme solution and 50 μL 50 mM citrate buffer (pH 4.5). After incubation at 50 °C for 30 min, 300 μL DNS was added to terminate the action, and the mixture was then maintained in 100 °C boiled water for 5 min. The absorption of the reaction mixture was measured at 540 nm using a Victor X3 Multimode Plate Reader (PerkinElmer, USA), whereby 1 unit (U) of enzyme activity produced 1 μmol glucose per minute in the reaction condition. Protein concentrations were determined via the manufacturer’s protocol using the Bradford Protein Assay Kit (Bicinchoninic acid method) (Tiangen Biotech (Beijing) Co. Ltd, China).

### Biochemical characterization of Cel-5A

The effect of pH on cellulase activity was determined by assaying for enzyme activity over the pH range of 3.0–10.0 using the following buffers: 50 mM citrate buffer with an interval of 0.5 (pH 3.0–5.0), 50 mM phosphate buffer with an interval of 1.0 (pH 6.0–8.0), and 50 mM glycine-NaOH with an interval of 1.0 (pH 9.0–10.0) at 50 °C for 30 min. For the pH stability assay, the enzyme was incubated at 50 °C in the different buffers for 30 min without substrate and subsequently measured for enzyme activity at 50 °C for 30 min.

The effect of the reaction temperature was measured between 30–100 °C (10 °C intervals) at the optimum pH for 30 min. The thermal stability of the enzyme was assessed at the forementioned temperature for 30 min, and the enzyme activity was then measured under the standard conditions.

The effect of metal ions (K^+^, Cu^2+^, Mg^2+^, Zn^2+^, Ca^2+^, Mn^2+^, Fe^2+^, Fe^3+^, Al^3+^, Co^2+^, Ti^4+^, Cs^+^, Li^+^, Ni^2+^, and Rb^2+^) and SDS at concentrations of 10 mM, 5 mM, and 1 mM, on enzyme activity after pre-incubation for 30 min at room temperature was measured under the standard conditions. The enzyme activity determined for the control was set as 100%, and the relative activity under each condition was recorded.

The kinetic parameters, *K*m (Michaelis–Menten constant) and *V*max (maximum velocity), were measured with the substrate (CMC-Na) between 2.5 and 20.0 mg/mL after incubation with the purified Cel-5A cellulase at pH 4.5 and 50 °C for 30 min. The data was plotted according to the Lineweaver–Burk method.

The substrate specificity of the purified Cel-5A was determined at 1% (w/v) with different substrates, including barley glucan, laminarin, pullulan, maltose, Avicel, xylan from beechwood, filter paper, and CMC-Na. The enzyme activity measured for CMC-Na was set as 100%, and the relative activity for each substrate was recorded.

### Sequence analysis and homology modeling

The obtained DNA sequence was translated into a protein sequence and analyzed by BLAST via the NCBI database. The phylogenetic tree of Cel-5A with other GH5 enzymes in different species was constructed via the neighbor-joining method using MEGA (v.6.0) and visualized with the iTOL website (https://itol.embl.de/). Multiple sequence alignments were completed using the Clustal Omega website (https://www.ebi.ac.uk/Tools/msa/clustalo/), and the signal peptide and related sequences were identified using the SignalP 5.0 server (http://www.cbs.dtu.dk/services/SignalP/). The molecular weight, theoretical pI, instability index, aliphatic index, and grand average of hydropathicity (GRAVY) were predicted using the website ExPASy website (https://web.expasy.org/protparam/). In addition, the secondary structure of the recombinant enzyme was predicted using online resources at the Bloomsbury Centre for Bioinformatics (http://bioinf.cs.ucl.ac.uk/psipred/)j, while the Cel-5A structure and its active sites were obtained via homology modeling using the RaptorX online server (http://raptorx.uchicago.edu/).

### Application of switchgrass and coffee grounds to saccharification

Lignocellulose switchgrass was crashed into a 40 mesh and the coffee grounds were washed using dilute water. Both were then dried overnight at 60 °C. The crude recombinant enzyme in the buffer (pH 4.5) was saccharified with 6% (w/v) switchgrass at 50 °C for 20 h. Three grades of crude enzyme concentration were set at 0%, 10%, 20%, and 40%. Reducing sugar content was measured from the hydrolyzation liquid after centrifugation via the DNS method. The percentage of saccharification was subsequently calculated as follows (Dadheech et al. 2018): Saccharification rate (%) = Sugar contents (mg)/Substrate (mg) × 100.

### Accession number

The accession number of strain *B. subtilis* 1AJ3 16S rRNA gene (No. MG062801) and endocellulase *CEL*-5A gene (No. MN795058) were submitted to the GenBank and accession numbers were obtained.

## Results

### Influence of culture medium pH on accumulating reducing sugar content of *B. subtilis* 1AJ3

The reducing sugar content in the cultural liquid at different initial pH levels demonstrates that strain 1AJ3 can accumulate a higher amount of reducing sugars under acidic conditions at pH 4.0 (Additional file 1). Moreover, it maintained a stable activity within pH values 4–9, and demonstrated the widest pH range in terms of enzyme activity.

### *CEL*-5A cloning, purification and sequence analysis

**Fig. 1:**
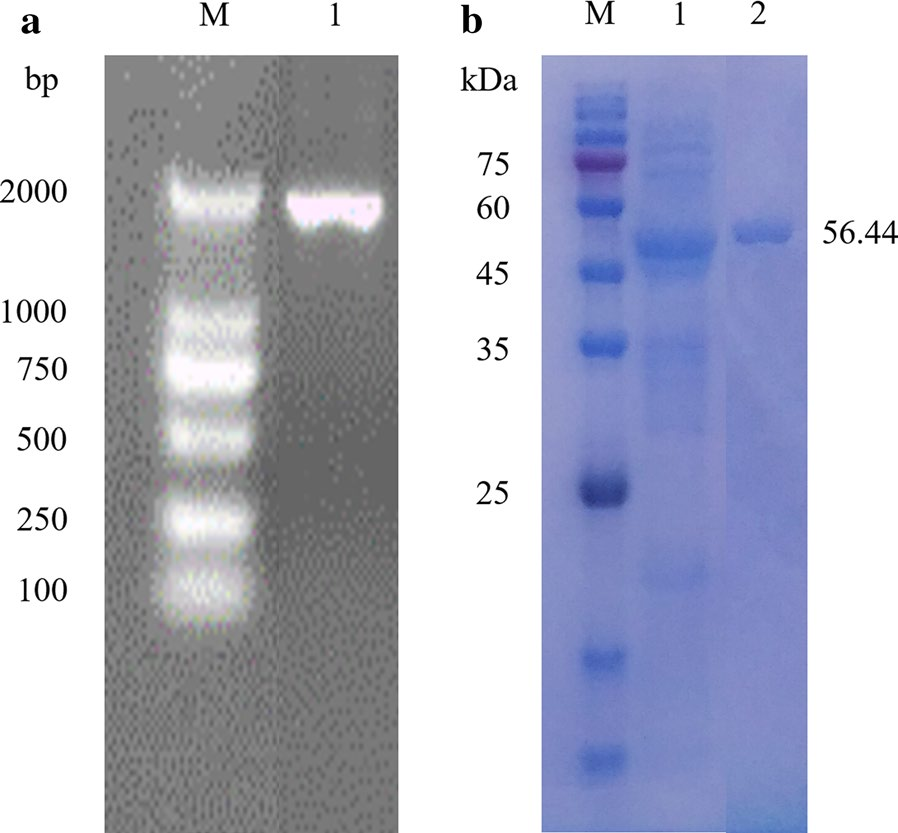
Agarose gel electrophoresis and SDS-PAGE of purified Cel-5A. **a** PCR product of Cel-5A recombinant plasmid by universal primers T7/T7er. **b** Expression and purification of Cel-5A

The whole ORF sequence of *CEL*-5A is shown in Fig. 1a. Different densities of IPTG were investigated in order to determine the maximum enzyme activity (Additional file 2). After being induced under 37 °C for 5 h with 0.2 mM IPTG, the soluble recombinant endo-cellulase Cel-5A was obtained. The recombinant enzyme was purified using Ni-NTA resin. The crude enzyme from the cell lysates supernatant and the purified enzyme were used with SDS-PAGE to detect the purification efficiency, with the molecular weight observed as 56 kDa (Fig. 1b).

**Fig. 2:**
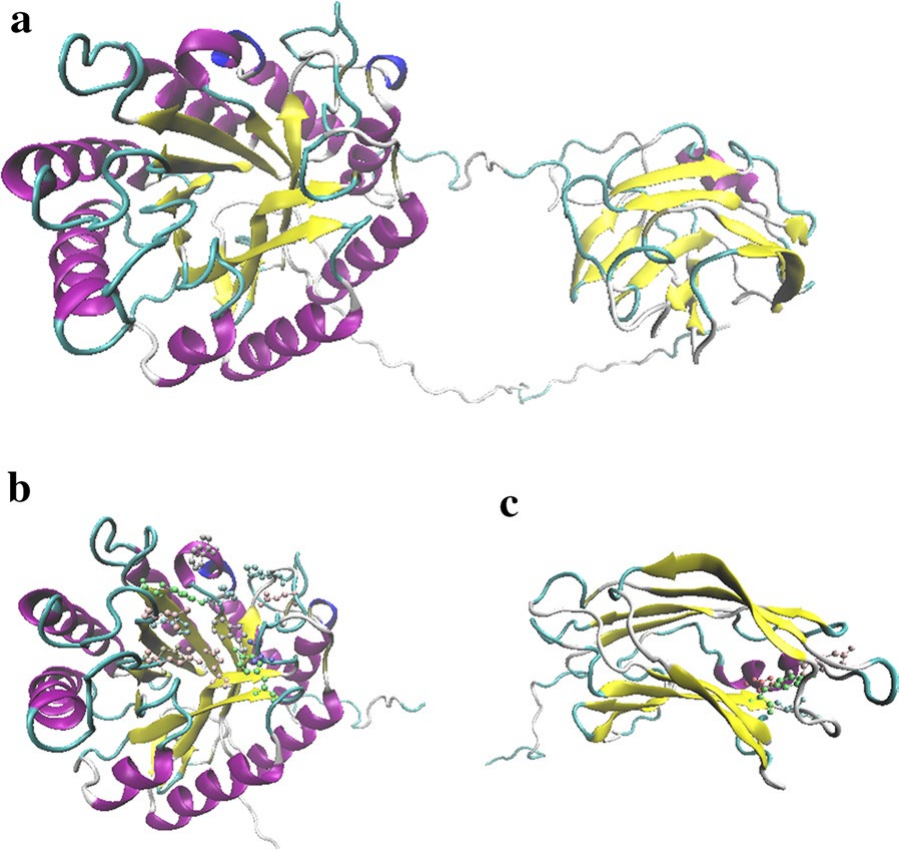
Homology modeling using the RaptorX online server for Cel-5A. **a** 3D structure of Cel-5A. **b** Active sites of the catalytic domain forming a pocket. **c** Active sites of the CBM domain

The theoretical pI and molecular weight were determined as 8.12 and 56.44 kDa, respectively. The SignalP 5.0 demonstrated the presence of a signal peptide on the N-terminal with a cleavage site between the Ala-29 and Ala-30. *CEL*-5A was observed to have two domains: (i) the catalytic domain (CD) from 1 to 351 amino acids, belonging to the GH5 family on the N-terminal; and (ii) the cellulose-binding domain (CBD) from 352 to 507 amino acid, belonging to the CBM3 family on C-terminal. The catalytic domain presented a classical TIM-like barrel motif with a (α/β)_8_ structure, while the CBM3 formed a beta sandwich with a jelly roll topology (Fig. 2a). The homology model indicated that CD was based on a 3D template structure of endo-1,4-beta-glucanase from *B. subtilis* 168 (PDB code 3PZT_A), with the identification of 97.48% by 62% query coverage of the whole amino acid sequence. Similarly, the CBM was based on a 3D template from a CBM3 lacking the calcium-binding site from *B. subtilis* 168 (PDB code 2L8A_A), with the identification of 99.32% by 28% query coverage. Differences in the amino acids sequences among the three domains are shown in Additional file 3. The Glu-169 and Glu-257 residues were conserved at a higher rate in the CD domain compared to the GH5 family. When the Cel-5A was aligned with the two models, differences were observed in the signal peptide from Met-1 to Ser-28, the linkage between the two domains from Lys-333 to Gly-353, and the amino acid Glu-465 site of CBM (Additional file 3: Fig S3). Active sites obtained from the RaptorX web server demonstrated that the CD domain forms an active pocket with the amino acid residues via His-65, Trp-69, Tyr-96, His-131, Leu-133, Asn-168, Glu-169, Tyr-231, Glu-257, Ala-263, Trp-291, Lys-296, and Glu-298 (Fig. 2b), and the CBD include active sites of Tyr-395, Lys-396, Glu-465, Gly-568, and Asp-469 (Fig. 2c).

**Fig. 3:**
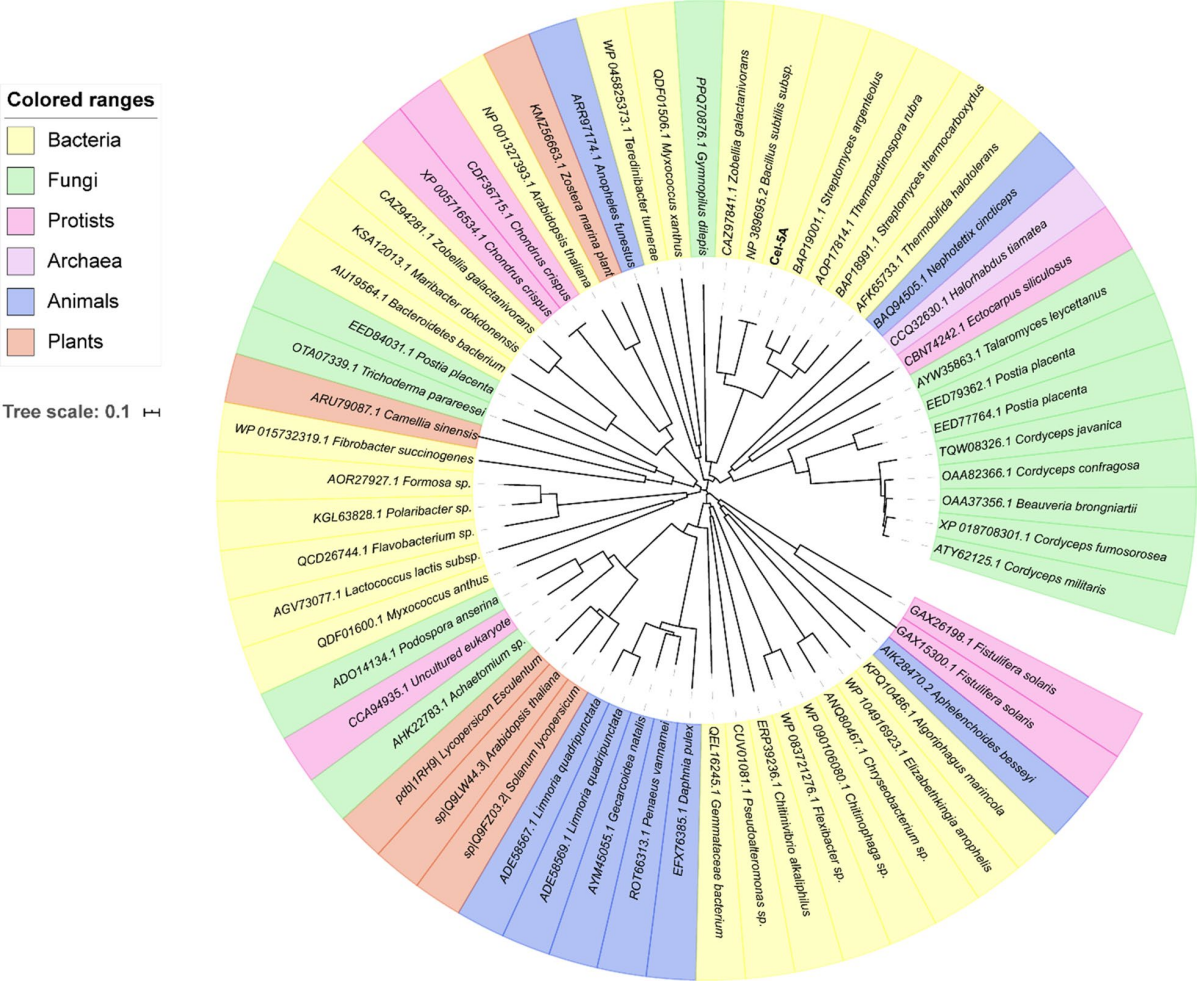
A neighbor-joining phylogenetic tree of the GH5 family cellulase of different species. Only the access number and species name are shown. Different colors represent different species: yellow for bacteria, green for fungi, pink for protists, purple for archaea, blue for animals, and red for plants

In order to gain a better understanding of the GH5 family cellulase in different species, neighbor-joining phylogenetic trees were constructed for bacteria, fungi, protists, archaea, animals, and plants (Fig. 3). It can be seen that the GH5 family is a large glucanase family with an extensive distribution variety.

### Characters of the recombinant Cel-5A enzyme

**Fig. 4:**
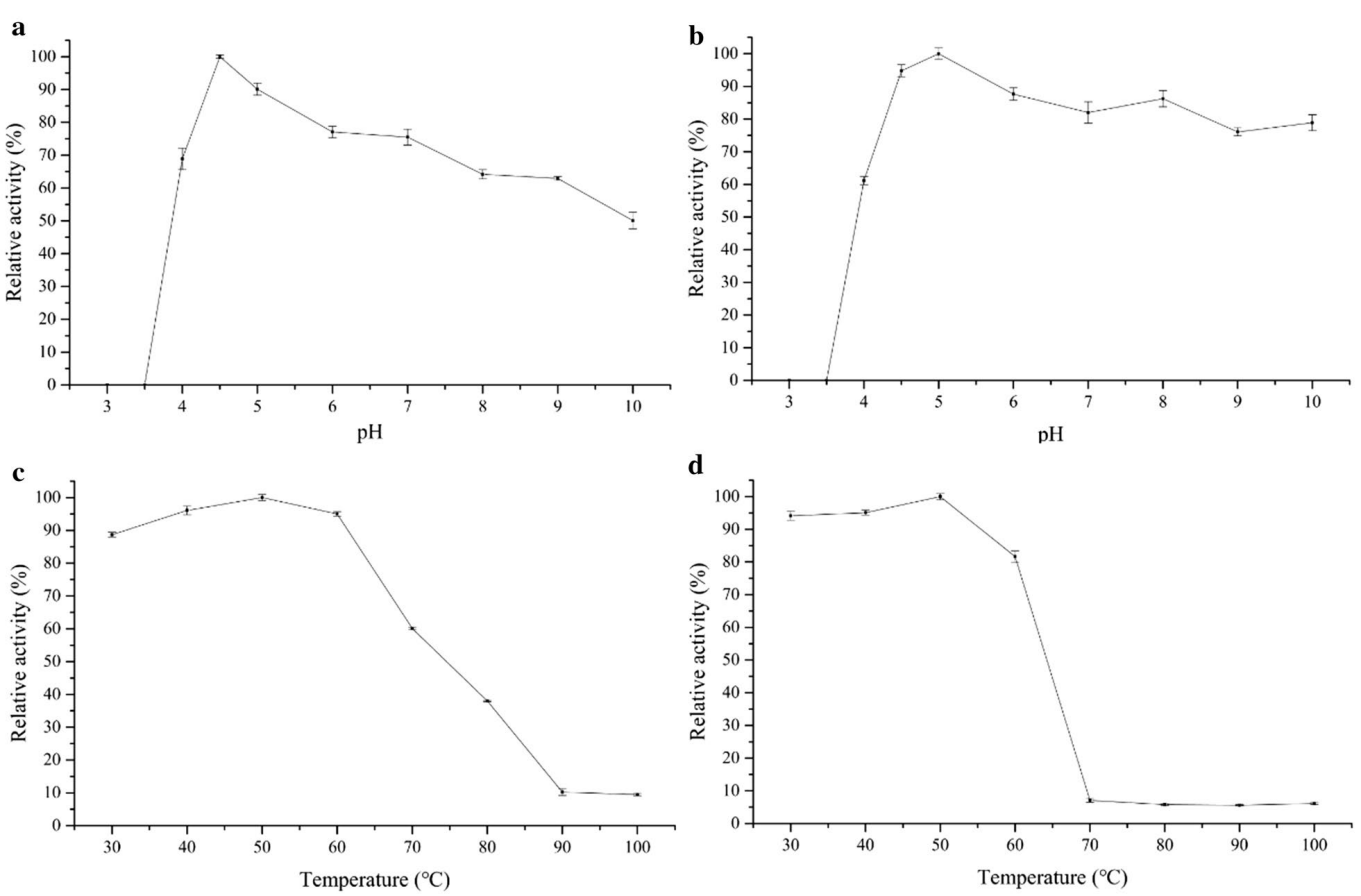
Effects of pH and temperature on the activity of recombinant Cel-5A. **a** Optimal pH. **b** Stable pH. **c** Optimal temperature. **d** Stable temperature

The activity of the purified recombinant enzyme Cel-5A was examined under different pH buffers ranging from 3.0 to 10.0 (Fig. 4a), to determine its optimum reactive pH level. The activity was observed to be at its highest at pH 4.5. Over 60% of maximum enzyme activity was attained from pH 4.0 to 9.0, signaling that Cel-5A can adapt to a wide pH region. For pH levels under 4.0, recombinant protein condensation became a precipitate, while a loss of activity was observed. pH stability levels indicated the optimum pH of Cel-5A lies between 4.5 and 10.0, with relative enzyme activity greater than 70%. Moreover, at pH 10, the enzyme activity was still high, with 78.86% relative activity (Fig. 4b). The results suggest that Cel-5A is stable over a wide pH range. The optimal temperature of Cel-5A was 50 °C, while it also demonstrated high activity levels between 30 and 60 °C (Fig. 4c). The activity of the enzyme decreased for temperatures greater than 70 °C, while for stable temperature, almost no enzyme activity was observed for 30 min (Fig. 4d). Cel-5A did not exhibit any thermostable characteristics under high temperatures, however a stable activity was observed under low temperatures. This demonstrates the potential application of Cel-5A with other enzymes with similar temperature related enzyme activity levels (e.g. laccase and manganese peroxidase) to hydrolyze lignocellulose.

The effect of metal ions on recombinant Cel-5A endo-cellulase activity is reported in Table 1. Co^2+^ and Fe^2+^ were able to significantly increase enzyme activity under the low concentration of 1 mM. Moreover, both high valence metal ions (Fe^3+^, Al^3+^, Ti^4+^) and Cu^2+^ were able to inhibit enzyme activity under high concentrations of 10 mM and 5 mM. However, low valency ions (Na^+^, K^+^, Cs^+^, Li^+^, Ni^+^, Mg^2+^, Zn^2+^, Ca^2+^, Mn^2+^, and Rb^2+^) had no observed effect on Cel-5A activity under high concentrations. Additionally, chemical SDS reduced the enzyme activity under high concentration levels and had no effect on activity under the concentration of 1 mM. The majority of ions at low concentrations demonstrated weak effects on cellulase activity, suggesting that Cel-5A has a wide ion range compatibility for industrial applications.

**Table 1:** Effect of metal ions and chemical on the cellulase activity of Cel-A5

Metal ion and chemicals	Relative activity (%)		
	10 mM	5 mM	1 mM
Na^+^	98.37 ± 0.72	98.67 ± 6.77	88.87 ± 0.92
K^+^	101.14 ± 0.69	94.08 ± 2.44	107.63 ± 3.93
Cu^2+^	15.77 ± 1.71	79.05 ± 0.54	115.23 ± 0.58
Mg^2+^	89.38 ± 3.07	88.74 ± 2.29	97.11 ± 0.63
Zn^2+^	85.71 ± 1.63	95.80 ± 3.35	119.36 ± 0.43
Ca^2+^	97.03 ± 1.02	88.44 ± 0.39	111.87 ± 0.57
Mn^2+^	74.25 ± 0.77	68.72 ± 2.40	73.91 ± 3.97
Fe^2+^	54.24 ± 0.37	106.77 ± 2.09	123.59 ± 0.67
Fe^3+^	3.91 ± 1.25	39.40 ± 6.74	99.16 ± 3.91
Al^3+^	1.78 ± 0.79	60.28 ± 0.67	104.19 ± 2.42
Co^2+^	110.15 ± 1.34	124.02 ± 5.36	140.38 ± 3.25
Ti^4+^	7.08 ± 0.24	18.85 ± 1.43	93.57 ± 1.07
Cs^+^	84.34 ± 0.83	92.50 ± 2.52	76.34 ± 2.56
Li^+^	91.89 ± 4.98	106.11 ± 2.12	76.84 ± 2.64
Ni^2+^	86.66 ± 3.12	105.32 ± 6.08	95.68 ± 1.47
Rb^2+^	85.18 ± 4.67	99.82 ± 1.52	99.61 ± 3.06
SDS	45.91 ± 0.90	29.04 ± 0.25	84.89 ± 1.65

Activity without any metal ion set as 100%

Substrate specific results (shown in Table 2) suggest that barley glucan was the most suitable Cel-5A substrate. Cel-5A was observed to affect β-1,3-1,4-gulcan linkages, but not α-glucan linkages. Low efficiencies were associated with the β-1,3, β-1,4, and β-1,6 glucan linkages. Moreover, the *K*m and *V*max Cel-5A values for CMC-Na were observed as 14.87 mg/mL and 19.19 μmol/min/mg, respectively.

**Table 2:** Substrate specificity analysis of recombinant enzyme and the original enzyme

Substrate (1%)	Glucan linkage	Relative activity (%)
Barley glucan	β-1,3-1,4 glucan linkage	168.47 ± 1.79
Laminarin	β-1,3 and β-1,6 glucan linkage	7.14 ± 0.12
Pullulan	α-1,4 and α-1,6 glucan linkage	ND
Maltose	α-1,4 glucan linkage	ND
Avicel	β-1,4 glucan linkage	11.01 ± 0.91
Filter paper	–	46.97 ± 0.88
Xylan from Beechwood	β-1,4 glucan linkage	13.95 ± 0.33
CMC-Na	–	100.00 ± 1.17

Depending on the substrate, activity was determined under optimal conditions. Enzyme activity using substrate of 1% CMC-Na was set of 100%

*ND* no detectable activity

### Potential application of Cel-5A in lignocellulose pretreatment

In order to further explore the application of Cel-5A in lignocellulose or biomass waste, lignocellulosic switchgrass was used as the substrate to detect the hydrolyzation station and saccharification abilities. In addition, a type of drinkable coffee ground was applied as the substrate to widen the application range. The crude enzyme was used for this portion of the experiment.

**Fig. 5:**
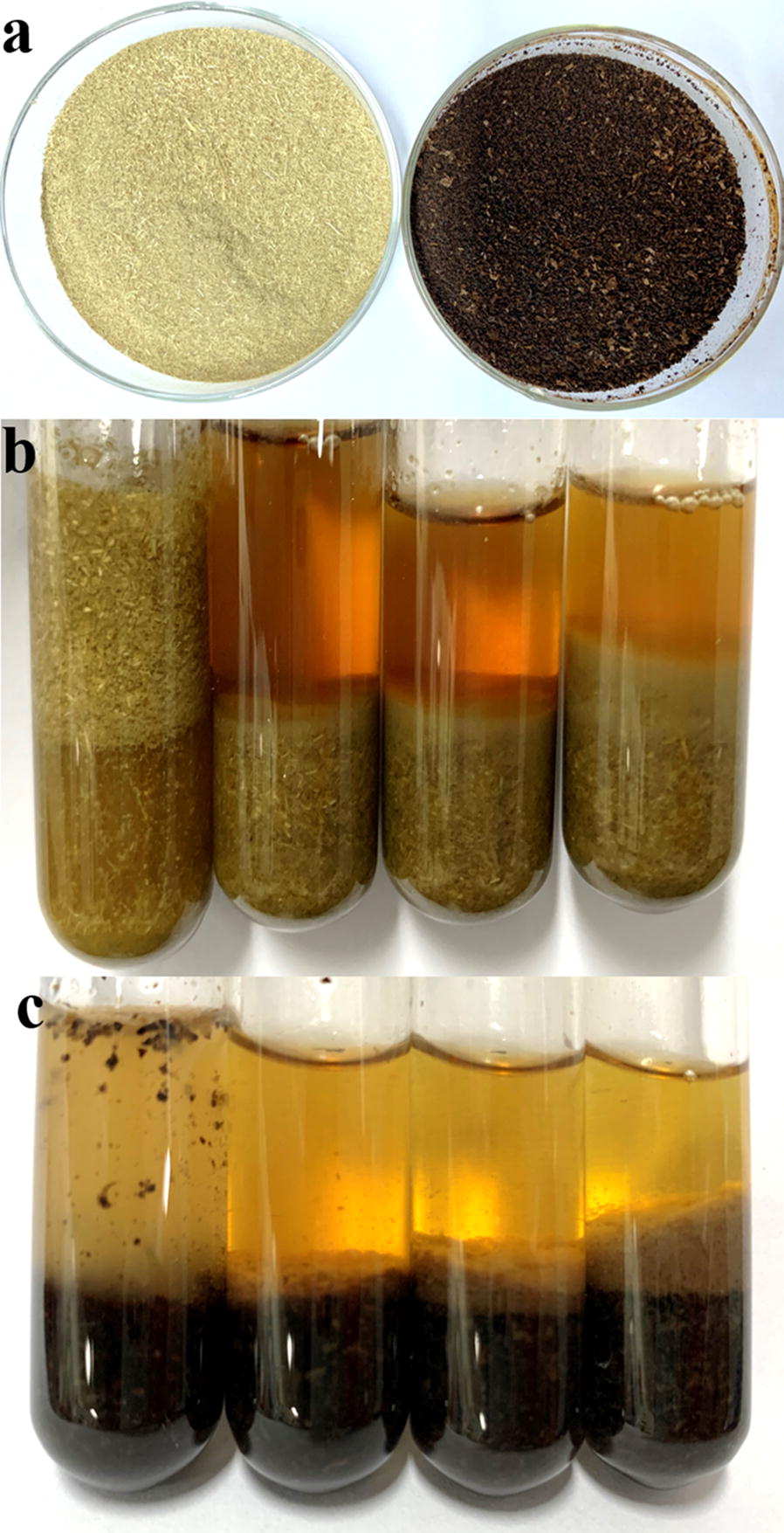
Recombinant enzyme applied in switchgrass and coffee grounds. **a** Switchgrass (left) and coffee grounds (right) raw materials following oven-drying. **b** Hydrolyzation of switchgrass by crude enzyme, with (left to right) the control, 10%, 20%, and 40%. **c** Hydrolyzation of coffee grounds by crude enzyme, with (left to right) the control, 10%, 20%, and 40%

After enzyme culturing for 20 h, the hydrolyzation phenomenon was evident, whereby the thick cellulose particles become fine and loose. This was particularly true for the coffee grounds, whereby the dark brown crushed granules turned into light-colored loose tiny particles (Fig. 5). The total reducing sugar content of switchgrass (coffee grounds) was observed with a saccharification rate of 12% (0.95%) among total weight. This demonstrates the high reducing sugar content of the switchgrass hydrolyzation production compared to that of the coffee grounds. Although coffee grounds can only produce reducing sugar at low levels, the hydrolyzation and degradation by Cel-5A also allow for the application of coffee wastes to the industry.

## Discussion

The development and utilization of cellulase plays a significant role in the new energy industry. Research on endocellulase must be continuously updated. Studies on cellulase and the determination of extreme enzyme characteristics are the focus of much research around the world, with the aim of identifying more applied enzymes for industrial usage. In current study, we cloned a GH5-CBM3 cellulase Cel-5A from a cellulolytic bacterium *B. subtilis* 1AJ3. As the largest hydrolysis family, the GH5 family, can hydrolyze cellulose independently, and is thus regarded as a catalytic domain. We can generally observe a cellulose-binding domain link to GH5, which is able to increase the combination ability between enzymes and cellulose. The majority of previous studies investigate the domains separately, with recent research make some domains together. However, the link between two independent domains will result in changes in the whole structure. In addition, the characteristics of the combination enzyme may differ within a single domain, while link peptides may modify the whole structure of the enzyme. Furthermore, the peptides between domains can also affect the characteristics (e.g. via their length or other specific property), thus influencing the structure and flexibility of the whole enzyme (Batista et al. 2011). In the current study, we characterized Cel-5A with two connecting domains, and explored the influence brought by the whole structure compared to those studied separately.

Since Cel-5A has high identification similarity of homographic sequence (separate CD and CBM domain) to *B. subtilis* 168, the GH5 and CBM3 domain cellulases have been studied separately in previous research (Santos et al. 2012), but we expressed the two domains together as a unity in the present study. Interestingly, Cel-5A with two domains combined display different characteristics to the case of separate domains. For example, the GH5 and CBM3 domains both exhibited thermophilic characters (Santos et al. 2012; Park et al. 2011; Lee et al. 2008) with an optimum or thermostable temperature at 80 °C, while this was not the case for Cel-5A. In the present study, the stable temperature of Cel-5A reached just 60 °C, with activity loss observed at 70 °C within 30 min. This may be attributed to the combination of the two domains, resulting in a change in the final enzyme structure, which subsequently alters the characteristics. Moreover, the similar scenario of a GH5 domain with low active temperatures between 25 and 55 °C has previously been reported (Yasir et al. 2013). Research has indicated that the low temperature stability of Cel-5A is triggered by the fall in the thermal stability in the whole enzyme of the presence of the CBM3 domain (Santos et al. 2012). In addition, two steps are required to convert cellulose into bioethanol, saccharification, and fermentation. A low temperature adaption can save energy and costs during the saccharification process. This can be degraded with low temperature enzymes (Tiwari et al. 2015; Khalili Ghadikolaei et al. 2018), and can thus be applied to a wide range of industries, such as the food, feedstuff, textiles, and pharmaceutical industries (Vester et al. 2014).

In terms of the pH, Cel-5A presented an acid-resistance ability, with pH 4.5 identified to optimize activity. Cel-5A activity was observed to be high within a wide pH range, with 62.9% of relative activity observed even at pH 9.0. In previous work, recombinant cellulase with the same hydrolyzation family domains (GH5 and CBM3) exhibited a different optimum pH region. The optimum pH for most recombinant cellulase with these domains has been observed as 4.5–4.7 (Park et al. 2011), while that of CelI15 as 6.0 (Yang et al. 2010), also with a GH5 CD and a CBM3 CBD, same families to those of this study. Higher optimum pH levels were observed for, for example, the same amino acids sequence GH5-CBM3 cellulase (pH 8.0) (Lee et al. 2008). The negative surface charge close to the active site could be the reason behind enzymes of lower pH stabilities (Dadheech et al. 2018).

Metal ions can lead to structural changes in enzymes, with the inhabitation and simulation of catalytic activities (Schiffmann et al. 2005). For most GH5 family cellulases, divalent metal ions (e.g. Fe^2+^, Cu^2+^, Zn^2+^) can inhibit enzyme activity (Feng et al. 2007; Voget et al. 2006). However, this contrasts to previous reports that suggest that a high concentration can inhibit activity, while low concentrations will not. The Co^2+^ ion was able to enhance enzyme activity, particularly at low concentrations (1 mM), with observed enhancements greater than those of Fe^2+^ ions. The same phenomenon was observed for by CelCM3 (Khalili Ghadikolaei et al. 2018). This can be attributed to the ability of Co^2+^ to form salt bridges in order to make enzyme structures more stable, thus increasing the enzyme activity (Correa et al. 2010). Mn^2+^ has previously been reported to exhibit a hyper-stabilizing effect on the GH5 family cellulase (Santos et al. 2012), however there was no observed improvement in the enzyme activity in the present study. Furthermore, the inhibitory effect of Mn^2+^ on Cel-5A activity agreed with previous research (Lee et al. 2008; Trivedi et al. 2011), due to the enhancement of the molecular rigidity, thus reducing enzyme activity (Gupta et al. 2017). In addition, studies have shown that Mn^2+^ has no effect on GH5 endoglucanases (Voget et al. 2006; Lee et al. 2014). The inhibition of Cel-5A via high concentrations of metal ions may be linked to the competition between exogenous and protein-associated cations, resulting in decreased metal-enzyme activity. This was particularly evident with Fe^3+^, Al^3+^ and Ti^4+^. Moreover, SDS as a detergent was able to drastically reduce the Cel-5A activity at high concentrations. Low-valent metal ions had almost no inhibition effects on enzymes activity, and employing Cel-5A in the industry is promising as the low concentration ions in hydrolyze-environment have no impact on the enzymes. Results also highlighted that mental ion concentrations in biomass hydrolyzation need to be examined cautiously when Cel-5A or other enzymes are to be supplemented.

For different carbon source substrates, the GH5 family cellulases generally demonstrated the capacity to hydrolyze both cellulosic and non-cellulosic substrates, acting on the β-1,4 linkage (Henrissat 1991). Furthermore, Cel-5A was highly hydrolyzed on CMC-Na and Barley β-glucan, but exhibited a low hydrolyzation ability on Avicel and Xylan from beechwood. Some GH5 cellulase was able to hydrolyze Avicel, while others were resistant to this substrate (Duan et al. 2009). Cel-5A was observed to be lightly resistant on Avicel, most likely because of the presence of the carbohydrate binding module (CBM) (Colussi et al. 2011; Gupta et al. 2017). The addition of CBM3 was able to help increase GH5 affinity (Maharjan et al. 2018), and enhance CD activity by creating a stronger bond between the enzyme and substrate. The observed *K*m value of Cel-5A for CMC-Na was 14.87 mg/mL, due to the greater level of affinity compared to GH5 and the GH5 family combined with CBM.

The hydrolyzation of unpretreated switchgrass and coffee grounds using Cel-5A demonstrated that enzyme hydrolyzation can be implemented as a pre-treatment of biomass materials, providing a potential utilization for switchgrass, coffee grounds, and cellulosic material waste. The biomass was observed to become looser even degrade. The results of the reducing sugar content highlighted the potential of switchgrass as a raw material for industrial ethanol. As Cel-5A is an endo-type enzyme, it can cleave cellulose chains of internal β-1,4-glucan linkages, thus converting biomass into shorter cellulose chains (Huy et al. 2016).

In a conclusion, in the current study, a two-domain GH5-CBM3 endocellulase was successfully cloned and heterologous expressed from a celluloytic *B. subtilis* 1AJ3. The characteristics of Cel-5A with two combined domains was different to those of separate domains. Its activity was observed within the wide pH range of 4.5-9.0 and low temperatures between 30-60 °C. The increased activity and stability of Cel-5A in the presence of various metal ions and its applicability in hydrolyze switchgrass and coffee grounds make it an attractive biocatalyst for the biomass and new energy industries. Further investigation must be performed on crystal structure analysis in order to explain the characteristics of Cel-5A.

## Supplementary information

**Additional file 1: Fig. S1.** Reducing sugar content of strain 1AJ3 cultural liquid after 48h under different initial pH.

**Additional file 2: Fig. S2.** Enzyme activity of *Cel*-5A by different IPTG density.

**Additional file 3: Fig S3.** Difference of amino acids between Cel-5A with two templets after align. 3PZT_A for GH5 and 2L8A_A for CBM3.

## Data Availability

The data on which the conclusions are made are all presented in this paper.
