# Supplementary material for: Cloning and characterization of low-temperature adapted GH5-CBM3 endo-cellulase from Bacillus subtilis 1AJ3 and their application in the saccharification of switchgrass and coffee grounds
Source: AMB Express. 2020 Mar 5;10:42. doi: 10.1186/s13568-020-00975-y (PMC7058755; doi:10.1186/s13568-020-00975-y)
Supplement: Supplementary file 1 — Additional file 1: Fig. S1. Reducing sugar content of strain 1AJ3 cultural liquid after 48h under different initial pH. [file 13568_2020_975_MOESM1_ESM.docx]

Additional file 1

Fig. S1 Reducing sugar content of strain 1AJ3 cultural liquid after 48h under different initial pH.
