# Supplementary material for: Cloning and characterization of low-temperature adapted GH5-CBM3 endo-cellulase from Bacillus subtilis 1AJ3 and their application in the saccharification of switchgrass and coffee grounds
Source: AMB Express. 2020 Mar 5;10:42. doi: 10.1186/s13568-020-00975-y (PMC7058755; doi:10.1186/s13568-020-00975-y)
Supplement: Supplementary file 2 — Additional file 2: Fig. S2. Enzyme activity of Cel-5A by different IPTG density. [file 13568_2020_975_MOESM2_ESM.docx]

Additional file 2

Fig. S2 Enzyme activity of *Cel*-5A by different IPTG density.
