# Supplementary material for: Cloning and characterization of low-temperature adapted GH5-CBM3 endo-cellulase from Bacillus subtilis 1AJ3 and their application in the saccharification of switchgrass and coffee grounds
Source: AMB Express. 2020 Mar 5;10:42. doi: 10.1186/s13568-020-00975-y (PMC7058755; doi:10.1186/s13568-020-00975-y)
Supplement: Supplementary file 3 — Additional file 3: Fig S3. Difference of amino acids between Cel-5A with two templets after align. 3PZT_A for GH5 and 2L8A_A for CBM3. [file 13568_2020_975_MOESM3_ESM.docx]

Additional file 3


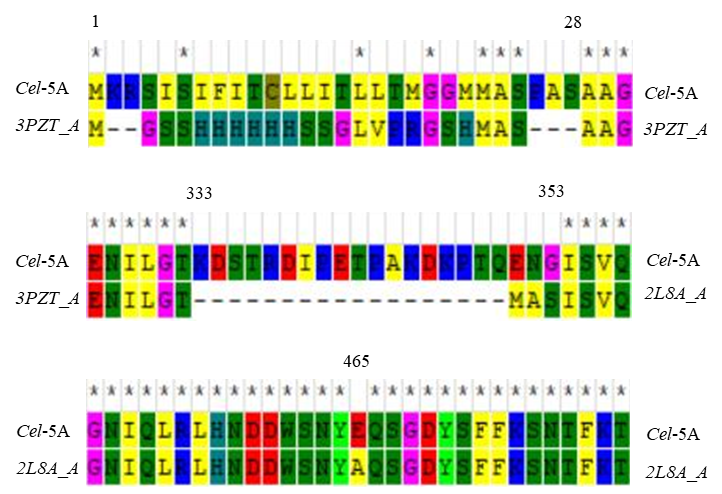


Fig S3. Difference of amino acids between Cel-5A with two templets after align. 3PZT_A for GH5 and 2L8A_A for CBM3.
